# An ecological analysis of walkability and housing affordability in Canada: Moderation by city size and neighbourhood property type composition

**DOI:** 10.1371/journal.pone.0285397

**Published:** 2023-05-31

**Authors:** Chelsea D. Christie, Christine M. Friedenreich, Jennifer E. Vena, Dany Doiron, Gavin R. McCormack

**Affiliations:** 1 Department of Community Health Sciences, Cumming School of Medicine, University of Calgary, Alberta, Canada; 2 Department of Cancer Epidemiology and Prevention Research, Cancer Care Alberta, Alberta Health Services, Calgary, Alberta Canada; 3 Alberta’s Tomorrow Project, Cancer Care Alberta, Alberta Health Services, Calgary, Alberta, Canada; 4 Research Institute of the McGill University Health Centre, Respiratory Epidemiology and Clinical Research Unit, Montréal, Quebec, Canada; 5 School of Architecture, Planning and Landscape, University of Calgary, Calgary, Alberta, Canada; 6 Faculty of Kinesiology, University of Calgary, Calgary, Alberta, Canada; 7 Faculty of Sport Sciences, Waseda University, Tokorozawa, Japan; University of Saskatchewan, CANADA

## Abstract

The neighbourhood built environment can support the physical activity of adults regardless of their individual-level socioeconomic status. However, physical activity supportive (walkable) neighbourhoods may not be accessible to those with lower incomes if homes in walkable neighbourhoods are too expensive. The objectives of this study were: 1) to estimate the associations between neighbourhood walkability and home values in Canadian cities, and 2) to test whether these associations differ by city size and residential property type composition within neighbourhoods. We linked built environment data from the 2016 Canadian Active Living Environments (Can-ALE) index with neighbourhood-level structural home characteristics and sociodemographic data from the 2016 Canadian census for 33,026 neighbourhoods across 31 Census Metropolitan Areas. We used multilevel linear regression models to estimate covariate-adjusted associations between neighbourhood walkability and natural-log median home values and tested city size and neighbourhood property type composition as moderators. There were no statistically significant associations between walkability and home values overall. The associations between neighbourhood walkability and home values were jointly moderated by city size and property type composition. For small and medium sized cities, within neighbourhoods containing a high proportion of detached homes, walkability was negatively associated with home values (*b* = -0.05, 95% CI: -0.10, -0.01; and, *b* = -0.04, 95% CI: -0.06, -0.02, for small and medium cities, respectively). However, for extra-large cities, within neighbourhoods containing a high proportion of detached homes, walkability was positively associated with home values (*b* = 0.06, 95% CI: 0.01, 0.10). Our findings suggest that, based on housing affordability, higher walkable neighbourhoods are likely accessible to lower income households that are situated in small and medium Canadian cities. In larger cities, however, municipal interventions (e.g., inclusionary zoning or targeted development of subsidized or social housing) may be needed to ensure equitable access to walkable neighbourhoods for lower income households.

## Introduction

Physical activity is undertaken in different settings (e.g., neighbourhoods, workplace, and recreational facilities) and for different purposes (e.g., leisure, exercise, or transportation) [1, 2]. Neighbourhoods, in particular, are common settings where adults accumulate much of their physical activity [3]. The built environment refers to any features of the physical environment that were created or modified by humans, including street design and land uses (e.g., commercial or residential) in a neighbourhood [4]. A substantial amount of evidence suggests that neighbourhood built environments can support walking [5, 6]. Findings from systematic reviews show that neighbourhood built environment characteristics including connectivity, land use mix and density (including the availability of recreational and utilitarian destinations), vegetation or greenness, population or residential density, and overall walkability (a composite measure that combines several built characteristics) are positively associated with physical activity [7–10].

Associations between the built environment and physical activity have been found in the Canadian context [7, 11–13], however, few of these studies have focused on socio-economically disadvantaged populations [14]. A recent systematic review identified only seven Canadian-based studies that examined associations between neighbourhood built characteristics and physical activity amongst adults with low socioeconomic status (SES) [14]. Despite the limited number of studies, residing in a walkable neighbourhood appears to provide similar support for physical activity for adults regardless of their SES [15, 16]. Yet, while the neighbourhood built environment can support physical activity even among low SES adults, low SES adults may have limited access to these physical activity supportive neighbourhoods (i.e., via home ownership or renting), if there is a lack of affordable housing [17–19].

Several Canadian studies have examined associations between the built environment and neighbourhood SES, home values (estimated by homeowners or from property taxes), or home prices (from real estate transactions). Canadian studies examining access to physical activity supportive neighbourhoods among samples with low SES have found mixed results. For instance, favourable built environment characteristics such as high levels of walkability and greenness were found to be less prevalent in low SES neighbourhoods within Montréal, Toronto, and Vancouver [20] and higher levels of walkability were associated with higher home prices in Calgary [21]. By contrast, in Halifax, higher prices were found for homes situated in cul-de-sacs versus on streets that were part of a grid-pattern [22], suggesting a negative association between street connectivity (commonly associated with walkability) and home prices. Finally, for public transportation, the introduction of a rapid bus transit system in Quebec City was associated with increases in nearby detached home prices [23], but proximity to bus stops was negatively associated with home prices of detached and non-detached homes in Calgary [21].

To date, most Canadian studies examining associations between neighbourhood walkability with neighbourhood SES or with home values have been undertaken in larger, more populated, cities. Differences in affordability of homes and desires to reside in physical activity supportive neighbourhoods may be driven by high demand and low supply within certain real estate markets. Cities such as Toronto, Vancouver, Ottawa, and Calgary have experienced booms in real estate markets during recent decades where excessively high demands fueled upwardly spiraling home costs [24]. These real estate booms may lead to increased geographical segregation by income within larger cities that may not be found within smaller cities. This pattern has been referred to as suburbanization of poverty within large cities, including Toronto and Calgary [25–27]. Suburban areas and sprawling communities tend to have lower walkability [28] and have been linked to lower levels of physical activity [29, 30]. Therefore, it is important to consider whether city size moderates the association between neighbourhood walkability and housing affordability.

The impact of physical activity supportive built environments on home values may also differ by the type of property. In Austin (Texas, US), increases in walkability were found to be associated with higher home prices for condominiums [31], but not for detached homes [32]. Similarly, proximity to the San Diego light rail system in California (US), had a stronger positive association for condominium home prices compared to single-family home prices [33]. In Calgary (Alberta, Canada), for both detached and non-detached homes (e.g., townhouses and condominiums), positive associations were found between neighbourhood walkability and home prices; however, negative associations were found between proximity to a bus stop and home prices [21]. In contrast, population density had a positive association with the prices of non-detached homes, but a negative association with the prices of detached homes in Calgary [21].

Encouraging adults to walk more is important because walking is associated with numerous health benefits [34–36] and can be undertaken by most adults regardless of their demographic characteristics and socioeconomic circumstances [37, 38]. However, adults with lower SES tend to be less physically active than adults with higher SES [39–41]. Estimates from the Canadian Community Health Surveys (2015–2018) indicated that the prevalence of physical inactivity was 34% among the highest income quintile, but 51% in the lowest income quintile [42]. Low SES adults residing in more walkable neighbourhoods benefit from increased levels of physical activity [15, 16], however, inequitable access to homes in walkable neighbourhoods may negatively impact opportunities for physical activity and contribute to health inequities in low SES households.

The objectives of this study are two-fold: 1) to estimate the associations between neighbourhood walkability and home values in Canadian cities, and; 2) to determine whether these associations differ by city size and by the proportion of different residential property types (i.e., detached or non-detached homes) in the neighbourhood. We hypothesize that higher neighbourhood walkability is associated with higher home values and that these associations will differ based on city size and neighbourhood composition of housing types. Specifically, we hypothesize that neighbourhood walkability will have a stronger positive association with home values in neighbourhoods with a low proportion of detached homes in the neighbourhood. Due to limited prior research, we have no directional hypotheses related to moderation by city size or joint moderation by city size and residential property type.

## Methods

### Sample

The sample for this cross-sectional ecological study consisted of neighbourhoods within medium-to-large cities in Canada. Canadian cities were included if they met the criteria for a Census Metropolitan Areas in 2016 and had transit data available for the Can-ALE walkability index. Census Metropolitan Areas (CMA) refer to cities in Canada with a total population of at least 100,000, with 50,000 or more people living in the core [43]. Neighbourhoods were operationalized as dissemination areas (DA), a geographic unit defined by Statistics Canada that typically encompasses a population of between 400 and 700 people [44]. Esri (Environmental Systems Research Institute) ArcGIS Desktop version 10.7 was used to clip target DAs from within CMA Shapefiles corresponding to the 2016 Canadian Federal Census. Neighbourhood-level structural home data and sociodemographic data from the 2016 Canadian Census were linked to built environment data from the 2016 Canadian Active Living Environments (Can-ALE) index [45, 46]. The University of Calgary Conjoint Research Ethics Board approved this study (REB21-0583) and the need for consent was waived by the ethics committee.

### Variables

#### Walkability

The 2016 Canadian Active Living Environments (Can-ALE) index was obtained from the Canadian Urban Environmental Health Research Consortium (CANUE) [47] and used as a measure of walkability of the neighbourhood. The Can-ALE index consists of intersection density, dwelling density, points of interest, and transit stops, and was calculated using a 1 km circular buffer around the center of each DA [46]. Intersection density was estimated using road and footpath features from OpenStreetMap and represents the number of three-way (or more) intersections within the 1 km buffer. To focus on active transportation routes, the intersection density measure included offroad footpaths/recreational trails and excluded highways [46]. Dwelling density consisted of the average dwelling density within the 1 km buffer area using Statistics Canada census data. The points of interest measure (derived using OpenStreet Map) included the number of destinations (e.g., parks, schools, shops, places of business and landmarks) within a 1 km buffer. The transit stops measure was estimated from the number of public transit stops or stations in the 1 km buffer around a dissemination area centroid using transit data obtained from websites of transit agencies and/or municipalities [46]. The Can-ALE index was estimated based on the sum of z-scores of the four built characteristics and represents a measure of walkability relative to DAs in Canadian CMAs [46]. Possible Can-ALE scores range from -3.55 to 47.83.

#### Median neighbourhood home values

As an indicator of housing affordability, household-level self-report data collected in the Canadian Census were aggregated to the DA-level to estimate the neighbourhood median home value. Respondents of owner-occupied, non-farm, private dwellings were asked in the 2016 long-form census: “If you were to sell this dwelling now, for how much would you expect to sell it?” Median home values were estimated for each DA and then natural log-transformed to meet the assumption of normality for linear regression modeling. Aggregate neighbourhood home value data have been used elsewhere when examining the relationship between walkability and home values [48].

#### Covariates

We selected covariates that were available in our dataset and that had the potential to be associated with neighbourhood walkability and home values. The distribution of structural characteristics of homes and sociodemographic characteristics within each neighbourhood (i.e., DA), available from the Statistics Canada 2016 census, were estimated as either means, medians, or proportions and included as covariates. Proportion variables had categorical response options that were mutually exclusive, thus values above 1 were assumed to be errors and truncated to 1 (n = 1440, across 7 variables). Proportion of dwellings in good condition within the neighbourhood was the proportion variable with the largest number of proportion estimates above 1 (n = 594) and had the highest maximum value truncated (1.08), possibly because some participants incorrectly selected two of the three options in response to the question “is this dwelling in need of any repairs” (e.g., “no, only regular maintenance is needed” and “yes, minor repairs are needed). Neighbourhood structural home characteristics included: mean number of rooms per dwelling, proportion of homes under five years of age, proportion of single-detached homes, and proportion of dwellings in good condition. Neighbourhood sociodemographic characteristics included: median household income, proportion of homeowners, proportion of non-visible minorities, proportion of non-immigrants, and proportion of non-movers within the last five years.

#### Potential moderators

Informed by previous findings [21, 33], two potential moderators were examined in relation to walkability and home values: residential property type (i.e., proportion of detached homes in the neighbourhood) and city population size. Data from the Statistics Canada 2016 census were used to determine the proportion of detached homes in the neighbourhood (count of single detached dwellings divided by the total count of dwellings). Census collection representatives recorded property type during home visits [49]. In areas where questionnaires were mailed to respondents and no enumerator or canvasser visited the dwelling, the structural type of dwelling reflects the classification from the 2006 census [49]. We used the 2016 Statistics Canada Population Centre and Rural Area Classification 2016 [50] cut points and the 2016 Statistics Canada city population sizes [51] to create four city size categories (population centres between 30,000 and 99,999 inhabitants = small, 100,000 to 1 million inhabitants = medium, 1 million to 2 million inhabitants = large, and over 2 million inhabitants = extra large).

### Statistical analyses

Descriptive analyses were used to compare cities across neighbourhood structural characteristics, sociodemographic characteristics, built environment characteristics, and home values. For each city, we estimated Pearson’s correlation coefficients and partial correlation coefficients (adjusting for proportion of detached homes) between neighbourhood walkability and home values.

To estimate associations between neighbourhood walkability and home values, we used multilevel linear regression models using the lme4 package [52] in R, version 4.1.3. Random slopes models were used to allow the slope to vary by city and to allow the testing of cross-level city-size by neighbourhood walkability interactions. Neighbourhoods (level 1) were nested within cities (level 2). Level 1 predictors were centered on the mean for each city (i.e., group-mean centered) by subtracting the mean for the city from each neighbourhood-level predictor. We entered neighbourhood-level walkability and city-level walkability as separate predictors to parcel out within-city and between-city walkability effects on log median neighbourhood home values. Multilevel linear regression models were used to estimate beta coefficients (*b*) and 95% Confidence Intervals (CI) for neighbourhood walkability (as a random effect) and (log) median home values, while adjusting for mean city walkability, structural home characteristics, and sociodemographic covariates (as fixed effects).

To examine moderation by neighbourhood property type, a two-way interaction term for proportion of detached homes with neighbourhood walkability was included in the model as a random effect. To examine moderation by city size, a two-way interaction term (between walkability and city size) was included in the model as a fixed effect. To examine joint-moderation (proportion of detached homes and city size and walkability), we included a three-way interaction term in the model as a fixed effect.

## Results

### Sample characteristics

We excluded four CMAs (Belleville, Peterborough, Saguenay, and Trois-Rivières) because public transit data was unavailable. We removed 2200 DAs with missing data (6% of the sample), resulting in an analytic sample size of 33,026 DAs across 31 CMAs. The fewest number of DAs were in Lethbridge (n = 168) and the highest number in Toronto (n = 7035) (Table 1). The 2016 population counts for the CMAs ranged from 117,394 (Lethbridge) to 5,928,040 (Toronto) [51]. Five cities met the criteria for a small city, 20 for a medium city, three for large, and three for extra-large. The overall mean for the proportion of detached homes in a neighbourhood was 0.55 (SD = 0.35). The city with the lowest mean proportion of detached homes was Vancouver (0.40) and the city with the highest proportion was Regina (0.75). Walkability across all neighbourhoods ranged from -3.55 to 47.83, with a mean of 0.82 (SD = 3.64). For walkability across cities, Saint John had the lowest median walkability (-2.83), and Toronto had the highest (1.13) (Table 2). Median neighbourhood home values also varied, with an overall sample mean of $511,379 (SD = $389,879), with city-level median neighbourhood home values ranging from $160,148 in Moncton to $899,396 in Vancouver (Table 2).

**Table 1:** Neighbourhood (DA) structural characteristics and social characteristics, by city, Canadian CMAs with transit data, 2016.

City	Size*	DAs (n)	Proportion Detached Homes	Proportion Homes <5 Years Old	Proportion Good Condition	Mean Number of Rooms	Median Household Income	Proportion Home-Owners	Proportion Non-Minorities	Proportion Non-Immigrants	Proportion Non-Movers (5 years)
Toronto	XL	7,035	0.54	0.03	0.95	6.65	$89,856	0.77	0.56	0.55	0.67
Montréal	XL	5,929	0.41	0.03	0.93	5.93	$65,024	0.62	0.80	0.76	0.64
Vancouver	XL	3,300	0.40	0.07	0.94	6.24	$81,664	0.69	0.53	0.57	0.59
Ottawa	L	1,826	0.54	0.04	0.94	6.79	$92,566	0.74	0.82	0.81	0.65
Calgary	L	1,686	0.65	0.04	0.95	6.78	$99,328	0.75	0.71	0.71	0.58
Edmonton	L	1,565	0.66	0.05	0.94	6.73	$96,512	0.73	0.77	0.77	0.60
Québec	M	1,196	0.50	0.05	0.95	6.10	$72,192	0.67	0.96	0.94	0.67
Winnipeg	M	1,148	0.73	0.03	0.92	6.33	$77,312	0.76	0.78	0.77	0.64
Hamilton	M	1,142	0.66	0.03	0.94	6.92	$84,118	0.77	0.85	0.77	0.67
London	M	727	0.66	0.03	0.94	7.04	$71,552	0.73	0.88	0.82	0.64
Kitchener	M	703	0.61	0.03	0.95	6.81	$80,486	0.73	0.85	0.78	0.64
St. Catharines	M	670	0.71	0.03	0.93	6.72	$66,368	0.75	0.92	0.83	0.66
Oshawa	M	555	0.71	0.04	0.95	7.16	$91,520	0.82	0.85	0.82	0.67
Halifax	M	548	0.60	0.04	0.93	6.80	$75,968	0.70	0.91	0.90	0.64
Victoria	M	541	0.45	0.04	0.95	6.24	$77,312	0.66	0.86	0.79	0.58
Windsor	M	510	0.75	0.02	0.93	6.86	$73,920	0.76	0.82	0.78	0.66
Saskatoon	M	400	0.68	0.05	0.94	6.89	$85,350	0.73	0.85	0.85	0.58
Regina	M	363	0.75	0.04	0.92	6.73	$87,680	0.75	0.85	0.85	0.61
Barrie	M	324	0.72	0.03	0.95	7.16	$86,166	0.78	0.92	0.87	0.61
St. John’s	M	313	0.55	0.07	0.95	7.03	$81,459	0.71	0.96	0.94	0.65
Sherbrooke	M	296	0.51	0.06	0.95	6.02	$57,888	0.61	0.95	0.93	0.61
Sudbury	S	257	0.67	0.03	0.92	6.52	$77,568	0.72	0.97	0.94	0.66
Abbotsford	M	256	0.53	0.04	0.95	7.00	$83,853	0.74	0.74	0.76	0.59
Kelowna	M	231	0.58	0.05	0.95	6.95	$76,032	0.74	0.92	0.85	0.56
Kingston	M	231	0.65	0.03	0.94	6.82	$79,872	0.72	0.93	0.87	0.62
Thunder Bay	S	228	0.73	0.02	0.93	6.50	$69,888	0.75	0.96	0.90	0.69
Guelph	M	224	0.63	0.04	0.95	6.79	$86,634	0.74	0.85	0.80	0.63
Brantford	S	222	0.71	0.03	0.93	7.05	$77,120	0.76	0.94	0.88	0.66
Saint John	S	219	0.67	0.04	0.92	6.85	$69,632	0.77	0.96	0.95	0.69
Moncton	M	213	0.63	0.06	0.94	6.61	$65,536	0.72	0.95	0.94	0.64
Lethbridge	S	168	0.69	0.05	0.94	6.95	$72,064	0.70	0.89	0.85	0.58

Note. Cities are arranged by number of neighbourhoods (decreasing order). Proportion variables are mean proportion values for the neighbourhoods. Median income is the median of the median income values for neighbourhoods.

*City size categories are based on 2016 population size (between 30,000 and 99,999 inhabitants = small, 100,000 to 1 million inhabitants = medium, 1 million to 2 million inhabitants = large, and over 2 million inhabitants = extra large)

**Table 2:** Distributions of neighbourhood walkability and home values, by city, Canadian CMAs with transit data, 2016.

	Walkability				Home Values			
City	P25*	Median (rank)	P75*	Max Value	P25*	Median (rank)	P75*	Max Value
Toronto	-0.09	1.13 ^(1)^	2.94	47.83	$501,496	$651,247^(2)^	$849,288	$3,008,497
Montréal	-0.64	1.02^(2)^	3.41	19.34	$259,237	$319,993^(15)^	$401,214	$2,268,683
Winnipeg	-0.41	0.72^(3)^	1.88	12.12	$229,868	$285,334^(20)^	$348,206	$802,802
Calgary	-0.57	0.53^(4)^	1.53	15.10	$365,128	$449,031^(6)^	$552,284	$2,210,198
Ottawa	-1.46	0.47^(5)^	1.86	13.75	$275,043	$349,634^(12)^	$448,738	$2,002,140
Vancouver	-0.80	0.40^(6)^	2.16	28.88	$601,072	$899,396^(1)^	$1,346,988	$4,797,202
Victoria	-1.28	0.36^(7)^	1.82	8.95	$449,684	$596,043^(3)^	$698,925	$2,298,306
Hamilton	-1.17	0.23^(8)^	1.49	13.15	$320,264	$400,604^(8)^	$532,230	$1,500,953
Regina	-0.62	0.19^(9)^	0.66	3.59	$277,689	$319,659^(16)^	$379,582	$781,829
Edmonton	-1.28	0.02^(10)^	0.88	9.59	$323,735	$376,699^(10)^	$449,211	$1,002,149
Québec	-1.42	-0.04^(11)^	1.42	13.94	$249,877	$259,188^(23)^	$299,750	$897,342
Saskatoon	-1.46	-0.18^(12)^	0.56	3.75	$299,074	$349,424^(13)^	$400,604	$900,749
Kitchener	-1.31	-0.30^(13)^	0.66	7.18	$297,202	$340,867^(14)^	$400,130	$1,301,533
Oshawa	-1.54	-0.64^(14)^	0.06	4.92	$345,067	$400,889^(7)^	$501,080	$900,831
Abbotsford	-2.21	-0.67^(15)^	0.86	7.01	$400,598	$500,716^(4)^	$598,480	$1,297,306
Lethbridge	-2.57	-0.77^(16)^	-0.17	1.67	$250,356	$277,542^(21)^	$310,793	$698,186
London	-2.17	-0.81^(17)^	0.18	4.67	$198,850	$250,349^(26)^	$324,347	$751,155
Halifax	-3.16	-0.88^(18)^	0.96	9.68	$219,824	$266,308^(22)^	$341,210	$993,511
Guelph	-2.04	-1.00^(19)^	-0.46	3.18	$347,596	$400,464^(9)^	$500,137	$1,001,988
Kingston	-3.42	-1.02^(20)^	0.76	10.69	$250,811	$299,774^(18)^	$349,169	$802,017
St. Catharines	-2.36	-1.04^(21)^	-0.04	3.40	$199,722	$250,366^(25)^	$310,999	$698,967
Thunder Bay	-2.48	-1.08^(22)^	-0.10	1.34	$179,778	$225,026^(27)^	$278,753	$425,776
Sherbrooke	-3.06	-1.25^(23)^	0.80	5.45	$190,100	$200,539^(28)^	$250,199	$500,129
Brantford	-2.75	-1.40^(24)^	-0.35	2.26	$215,676	$299,166^(19)^	$349,999	$696,812
Barrie	-2.67	-1.42^(25)^	-0.72	1.29	$340,817	$376,508^(11)^	$422,628	$801,132
Windsor	-2.34	-1.44^(26)^	-0.34	2.65	$134,409	$190,069^(29)^	$259,477	$599,998
St. John’s	-2.94	-1.45^(27)^	0.15	4.29	$269,325	$300,232^(17)^	$348,854	$647,811
Moncton	-3.38	-2.01^(28)^	-0.88	2.78	$139,960	$160,148^(31)^	$194,840	$374,998
Kelowna	-2.90	-2.07^(29)^	-0.89	3.05	$399,676	$449,499^(5)^	$553,406	$1,500,552
Sudbury	-3.24	-2.23^(30)^	-0.33	8.91	$219,952	$250,369^(24)^	$295,270	$698,581
Saint John	-3.50	-2.83^(31)^	-1.58	4.60	$144,807	$170,747^(30)^	$200,646	$450,437

Note. Cities are arranged by median city-level walkability.

*P25 and P75 refer to the 25^th^ and 75^th^ percentiles.

The spearman rank correlation between median city-level walkability and median home values is 0.56 (95% CI: 0.25, 0.77).

### Associations between neighbourhood walkability and median home prices

Pearson correlations between neighbourhood walkability and log median home values ranged from -0.65 in Abbotsford to 0.34 in Montréal. After adjusting for the proportion of detached homes, 14 cities had negative, 10 cities had positive, and 7 cities had non-statistically significant correlations between neighbourhood walkability and home values (Supporting Information).

In the empty multi-level model, the intraclass correlation coefficient (ICC) was 0.47, indicating that a large proportion of variance in median neighbourhood home prices was accounted for by city-level factors (level-2). In unadjusted models, both within-city walkability (*b* = -0.05, 95% CI: -0.07, -0.03) and between-city walkability (*b* = 0.26; 95% CI: 0.17, 0.36) were associated with log median neighbourhood home values. After adjusting for neighbourhood structural home characteristics and sociodemographic characteristics, the mean association between neighbourhood-level walkability and log median home values was attenuated and no longer statistically significant (*b* = -0.01, 95% CI: -0.02, 0.00). Similarly, the overall mean association between city-level walkability and log median home values was no longer significant after adjustment for covariates (*b* = 0.14, 95% CI: -0.02, 0.31) (Table 3).

**Table 3:** Associations between walkability and home values (outcome = log of median neighbourhood home value).

	Fully adjusted model no interaction terms			_	Fully adjusted model with interaction terms		
	Estimates	95% CI	*p*		Estimates	95% CI	*p*
(Intercept)	12.58	12.22 – 12.94	<0.001		12.47	12.13 – 12.81	<0.001
Neighbourhood walkability	-0.01	-0.02 – 0.00	0.132		-0.02	-0.05 – 0.01	0.144
City (mean) walkability	0.14	-0.02 – 0.31	0.086		0.07	-0.06 – 0.20	0.287
City size (small = REF) Medium (M)	0.16	-0.17 – 0.49	0.336		0.21	-0.12 – 0.55	0.214
Large (L)	0.29	-0.23 – 0.81	0.282		0.37	-0.14 – 0.89	0.157
Extra Large (XL)	0.47	-0.22 – 1.16	0.181		0.72	0.09 – 1.35	0.024
Proportion homes <5 years old	0.55	0.51 – 0.58	<0.001		0.54	0.50 – 0.58	<0.001
Proportion detached homes	0.11	0.10 – 0.13	<0.001		0.19	0.00 – 0.37	0.046
Proportion good condition	0.22	0.15 – 0.28	<0.001		0.19	0.12 – 0.25	<0.001
Mean number of rooms	0.16	0.15 – 0.16	<0.001		0.15	0.15 – 0.16	<0.001
Median household income (per $10K)	0.06	0.06 – 0.06	<0.001		0.06	0.06 – 0.06	<0.001
Proportion homeowners	-1.08	-1.11 – -1.06	<0.001		-1.00	-1.03 – -0.98	<0.001
Proportion non-minorities	0.40	0.38 – 0.43	<0.001		0.42	0.39 – 0.45	<0.001
Proportion non-immigrants	-1.02	-1.06 – -0.98	<0.001		-1.00	-1.04 – -0.97	<0.001
Proportion non-movers (5 years)	0.37	0.34 – 0.41	<0.001		0.36	0.33 – 0.39	<0.001
Neighbourhood walkability*city size [M]					0.01	-0.02 – 0.04	0.528
Neighbourhood walkability*city size [L]					0.04	-0.01 – 0.08	0.120
Neighbourhood walkability*city size [XL]					0.06	0.01 – 0.11	0.011
Neighbourhood walkability*proportion detached homes					-0.06	-0.12 – 0.01	0.071
City size [M]*proportion detached homes					-0.04	-0.25 – 0.16	0.676
City size [L]*proportion detached homes					-0.10	-0.39 – 0.19	0.484
City size [XL]*proportion detached homes					0.11	-0.18 – 0.40	0.445
Neighbourhood walkability*city size [M]* proportion detached homes					0.01	-0.06 – 0.08	0.780
Neighbourhood walkability*city size [L]* proportion detached homes					0.02	-0.08 – 0.11	0.739
Neighbourhood walkability*city size [XL]* proportion detached homes					0.12	0.02 – 0.21	0.014

### Moderation by property type and city size

**Fig 1:**
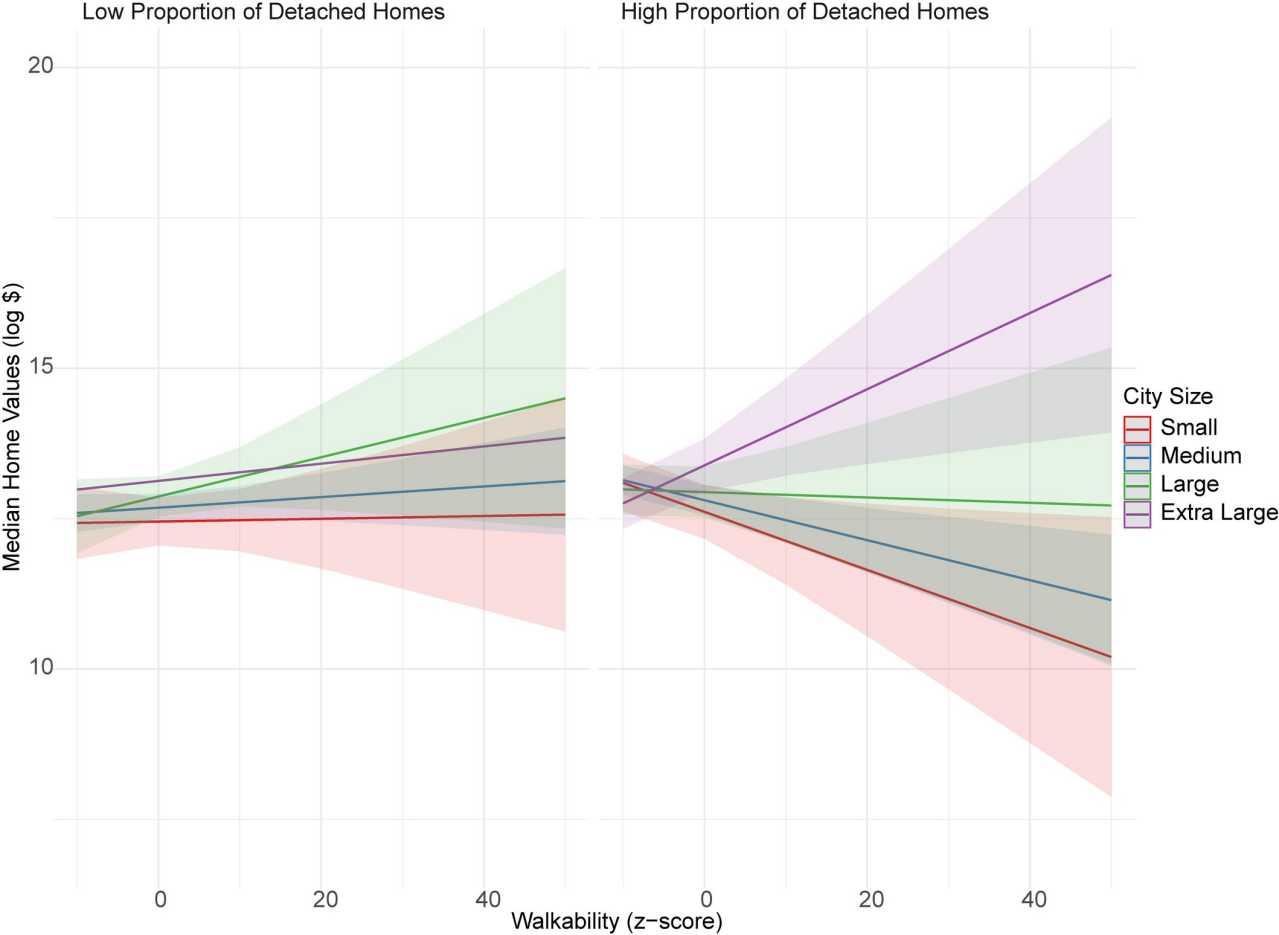
Joint moderation of median home values by proportion of detached homes and city size. Low and high proportions were determined using 10th and 90th percentiles of group-mean-centered proportion detached homes.

The association between neighbourhood walkability and home values was jointly moderated by residential property type and city size (p-value = 0.014, for the three-way interaction between walkability, extra-large city size, and proportion of detached homes). In other words, associations between neighbourhood walkability and home values were conditional on the size of the city and the proportion of detached homes within the neighbourhood (Fig 1). In small and medium sized cities, for neighbourhoods with a high proportion of detached homes, neighbourhood walkability was negatively associated with home values (*b*_small-city & high proportion of detached homes_ = -0.05, 95% CI: -0.10, -0.01; and, *b*_medium-city & high proportion of detached homes_ = -0.04, 95% CI: -0.06, -0.02, respectively). In contrast, in extra-large cities, for neighbourhoods with a high proportion of detached homes, neighbourhood walkability was positively associated with home values (*b*_extra large-city & high proportion of detached homes_ = 0.06, 95% CI: 0.01, 0.10). For other city size by housing type (high or low proportion of detached homes) combinations, the mean associations between walkability and home values were not statistically significant (Table 4). We also checked the models (separately) with only the two-way interaction terms included. In the absence of the three-way interaction term, the two-way interaction terms for walkability and city size were not statistically significant.

**Table 4:** Marginal effects estimates (slopes by city size and low or high proportion of detached homes; outcome = logarithm of median neighbourhood home value).

	Walkability Coefficients (95% Confidence Intervals)	
City Size	Low Proportion of detached homes	High proportion of detached homes
Small	0.00 (-0.04, 0.04)	-0.05 (-0.10, -0.01)*
Medium	0.01 (-0.01, 0.03)	-0.04 (-0.06, -0.02)*
Large	0.03 (-0.01, 0.08)	-0.01 (-0.06, - 0.04)
Extra Large	0.01 (-0.03, 0.06)	0.06 (0.01, 0.10)*

*p < 0.05

Note. Low and high proportion were determined using 10^th^ and 90^th^ percentiles of group-mean-centered proportion detached homes

## Discussion

Our study examined whether neighbourhood walkability was associated with home values across Canadian cities and whether these associations differed by city population size and by the proportion of residential property types (i.e., detached or non-detached homes) in the neighbourhood. Contrary to our hypothesis, after adjusting for neighbourhood-level structural home characteristics, sociodemographic characteristics, and city-level walkability, the overall mean association between neighbourhood-level walkability and home values across Canadian CMAs was not statistically significant. This finding is inconsistent with previous studies that have found positive associations between walkability and home values [21, 48, 53].

Notably, we found novel evidence suggesting that associations between neighbourhood walkability and home values might be conditional on the size of the city and the proportion of detached homes within the neighbourhood. For small- and medium-sized cities (populations of between 30,000 to 99,999 inhabitants or 100,000 to 1 million inhabitants, respectively), neighbourhood walkability was negatively associated with home values in neighbourhoods with a high proportion of detached homes. In contrast, in extra large cities (populations over 2 million inhabitants), neighbourhood walkability was positively associated with home values among neighbourhoods with a high proportion of detached homes. In neighbourhoods with a low proportion of detached homes, the associations between neighbourhood walkability and home values were not statistically significant, regardless of city size.

A similar study that focused on walkability and home prices (based on real estate transactions) in large US cities found results that are somewhat congruent with our findings. This study conducted separate linear regression models for each city and found that 13 out of the 15 cities had positive associations between neighbourhood walkability and home prices [53]. The cities in their sample had large populations (all over 600,000 inhabitants) and one of the three smaller cities in their sample (Bakersfield, CA) had a non-statistically significant association between walkability and home prices [53]. The higher proportion of positive associations found in their study may be explained by the higher number of large cities in their sample compared to our sample.

Importantly, our study is the first study examining associations between neighbourhood walkability and home values that also tested for joint moderation by city size and property type. For the joint moderation (by city size and property type) analysis of neighbourhood walkability and home values, our results are somewhat inconsistent with previous research. While other single-city studies have found a stronger positive association between neighbourhood walkability and home values for non-detached homes compared to detached homes [31–33], our national-level study found only statistically significant associations within neighbourhoods with a high proportion of detached homes. The moderating impact of property type was conditional on city size, which highlights the importance of examining this three-way interaction. The mix of differential associations by property type found in our study is similar to associations found between walkability and home prices in Calgary [21]. Specifically, Choi et al. [21] ran separate analyses for detached and non-detached homes and found similar results for neighbourhood walkability and proximity to a bus stop between the two property types. However, population density was positively associated with home values for non-detached homes and negatively associated with home values for detached homes [21].

The walkability-home value premium we observed in extra-large cities could be partially driven by a limited supply of houses within highly walkable areas [54]. Research in Toronto and Vancouver examining neighbourhood design preferences found an undersupply of compact, walkable, and transit-friendly neighbourhood types relative to current demand [55]. Another potential explanation of the walkability-home value premium in extra large cities could be the presence of additional amenities just beyond the 1km buffer, but still within walking distance. Future research that includes different buffer sizes could be useful for examining this hypothesis.

For small and medium-sized cities, the negative or null associations we found between walkability and home values may have been attributable to lower levels of gentrification or limited ranges of walkability levels. Inner cities tend to be more walkable [56] and also older (as cities tend to build outward [24]), which could indicate that inner city areas have older, less expensive housing. However, while inner cities of more populous cites may undergo gentrification, where newer, more expensive properties are built [57], we speculate that less gentrification is occurring in small to medium cities. Thus, for small to medium cities, the home value premium associated with newer homes in more peripheral areas may outweigh the potential benefits of being more centrally located. Another potential explanation for negative or null associations in small to medium cities is the limited range of neighbourhood walkability levels in these cities. Indeed, the majority of neighbourhoods in smaller cities within our sample had very low levels of walkability. Higher home values within more walkable neighbourhoods may only be more likely to occur when high quality transit (e.g., more direct routes and more route choice) is also available [56]. This hypothesized threshold effect could help explain the null associations found in smaller to medium cities, which tend to have less transit infrastructure and lower quality transit services (e.g., infrequent buses, fewer bus routes or less route coverage, and no commuter train or bus rapid transit system). Finally, the negative externalities associated with retail uses, such as noise, light, or trash [58] may not outweigh any potential benefits until high levels of walkability are reached. Similarly, although small and medium-sized cities have public transit, previous research has shown that access to transit may be associated with lower home values unless it is a rapid transit service [59].

A strength of this study is our inclusion of walkability and home value data from 31 of the 35 CMAs in Canada. Our estimates therefore reflect the overall (average) relationship between neighbourhood walkability and home values in CMAs across all of Canada. Previous research examining relationships between neighbourhood walkability and home values have typically focused on a single city with a relatively large population (e.g., Calgary or Halifax [21, 22]). Our study has extended this previous research by examining neighbourhood walkability and home values in a sample that included a range of cities that differed by both size and walkability. In addition, our use of home values as a primary outcome offers a useful policy-target as mortgage or rent payments constitute a major component of shelter costs. Shelter cost affordability has a strong influence on neighbourhood selection for low SES households. Another study strength is the use of a nationally-representative index of neighbourhood walkability (i.e., Can-ALE) that is correlated with physical activity [60] and with other health outcomes [61]. Moreover, the datasets used in our sample were temporally matched (i.e., collected in 2015/2016).

Despite its strengths, we acknowledge that our study has several limitations. Relying only on available data, our secondary analysis may have excluded potential confounders related to the built and social environmental characteristics of neighbourhoods (e.g., geographical distance to a city’s central business district, level of employment opportunities, pathway systems, greenspace, natural features, and crime rates) that are related to walkability and home values. Finally, home values were derived from a census question that was only answered by residents of owner-occupied homes. Thus, our results may not be generalizable to neighbourhoods that have very few owner-occupied homes (e.g., very low-income neighbourhoods).

There are a few implications of our findings. Contrary to our expectations, we did not find higher neighbourhood walkability to be associated with higher home values at a national level. Neighbourhood walkability in small to medium sized cities appears to have less impact on home values, and thus there is less concern with respect to perpetuating inequities in access to walkable environments in these cities. The exception, however, was within neighbourhoods with a high proportion of detached homes located in extra large cities (i.e., Toronto, Montréal, and Vancouver), where positive associations between neighbourhood walkability and home values were observed. Programs that assist in increasing the affordability of homes located in highly walkable neighbourhoods may be needed in large Canadian cities. Similarly, interventions to increase walkability of neighbourhoods in larger cities should also consider ways to ensure that lower income adults do not become priced out of these neighbourhoods [62]. Examples of such policies include the targeted development of affordable housing into neighbourhoods with high walkability and high-quality transit access [25, 63, 64] and/or the creation of mixed-income housing developments in walkable neighbourhoods, which would concurrently reduce the residential segregation of low-income families [65, 66].

Canada’s National Housing Strategy is an example of a policy that includes multiple components of these recommendations. A severe lack of affordable housing led to Canada’s first National Housing Strategy in 2017, which recognizes access to adequate housing as a human right. The Strategy expanded the commitment of federal and provincial governments to increase the social housing supply [67]. One component of the Strategy, the Rapid Housing Initiative, funds new projects that are required to meet a minimum threshold of affordability (tenants paying no more than 30% of their before-tax income on housing costs) and prioritizes projects that are mixed-tenure developments, mixed use developments, and/or where the housing will be located close to transit, employment, and recreational amenities [68].

Neighbourhood walkability and home value associations should continue to be monitored because of trends suggesting that the geographic patterning of home values is changing. In Canada, urban low-SES neighbourhoods have historically been centrally located [69], which also tend to be more walkable, but gentrification may be changing this pattern. In several large cities, such as Toronto, Montréal, Vancouver, and Calgary, research has demonstrated that inner-city communities are being transformed from being predominately low- to higher income areas, with a corresponding reduction of relative mean incomes in suburban neighbourhoods [26, 70]. These changing patterns of socioeconomic composition of people within neighbourhoods could increase or decrease home values and potentially alter how neighbourhood walkability is related to home values. Gentrification processes are further complicated by residents often advocating against changes to the built environment (e.g., increasing residential density) that could contribute to increased walkability. However, there is some evidence that the framing of messaging around increases in density can reduce these residents’ concerns [71, 72]. Future studies could include additional city-level predictors (e.g., inclusionary zoning policies) to examine ways cities can improve these neighbourhood walkability-home value associations. Future research should also examine associations between neighbourhood green space, parks, and cycling networks with home values.

In conclusion, we did not find evidence that more walkable neighbourhoods were less affordable in the majority of Canadian CMAs. The exception to this pattern was neighbourhoods in Toronto, Montréal, and Vancouver with a high proportion of detached homes, where there was a positive association between neighbourhood walkability and median home values. Differential associations by city-size emphasize the importance of examining the distinct contexts of different cities and points to the need for tailored interventions. It is important to keep evaluating socioeconomic equity in access to physical activity-supportive environments in case gentrification of higher walkability neighbourhoods continues to occur. Otherwise, the benefits of municipal urban planning and design strategies and policies intended to improve neighbourhood walkability may only benefit the physical activity and health of those who are able to afford housing in these more walkable neighbourhoods.

## Supporting information

S1 Table Correlations between walkability and (log) median home values, by city. (DOCX)
