# Supplementary material for: An ecological analysis of walkability and housing affordability in Canada: Moderation by city size and neighbourhood property type composition
Source: PLoS One. 2023 May 31;18(5):e0285397. doi: 10.1371/journal.pone.0285397 (PMC10231822; doi:10.1371/journal.pone.0285397)
Supplement: S1 Table — (DOCX) [file pone.0285397.s001.docx]

| **Table S1. Correlations between walkability and (log) median home values, by city** | | | | | | | | |
| --- | --- | --- | --- | --- | --- | --- | --- | --- |
| Pearson (bivariate) correlation between neighbourhood walkability and (log) median home values | | | |  | Partial correlation between neighbourhood walkability and (log) median home values, adjusting for proportion of detached homes | | | |
| City | *r* | 95% CI |  |  | City | *(partial) r* | 95% CI |  |
| Abbotsford | -0.65 | (-0.71, -0.57) |  |  | Abbotsford | -0.41 | (-0.51, -0.30) |  |
| Barrie | -0.37 | (-0.46, -0.27) |  |  | Barrie | -0.14 | (-0.25, -0.03) |  |
| Brantford | -0.60 | (-0.68, -0.51) |  |  | Brantford | -0.48 | (-0.57, -0.37) |  |
| Calgary | -0.20 | (-0.25, -0.16) |  |  | Calgary | 0.01 | (-0.04, 0.06) |  |
| Edmonton | -0.27 | (-0.32, -0.23) |  |  | Edmonton | -0.01 | (-0.06, 0.03) |  |
| Guelph | -0.37 | (-0.48, -0.25) |  |  | Guelph | -0.16 | (-0.28, -0.03) |  |
| Halifax | 0.30 | (0.22, 0.37) |  |  | Halifax | 0.45 | (0.38, 0.52) |  |
| Hamilton | -0.45 | (-0.50, -0.41) |  |  | Hamilton | -0.36 | (-0.41, -0.31) |  |
| Kelowna | -0.53 | (-0.62, -0.43) |  |  | Kelowna | -0.09 | (-0.22, 0.04) |  |
| Kingston | 0.13 | (0.00, 0.26) |  |  | Kingston | 0.37 | (0.25, 0.48) |  |
| Kitchener | -0.35 | (-0.42, -0.29) |  |  | Kitchener | -0.14 | (-0.21, -0.07) |  |
| Lethbridge | -0.38 | (-0.5, -0.24) |  |  | Lethbridge | -0.24 | (-0.38, -0.09) |  |
| London | -0.33 | (-0.4, -0.27) |  |  | London | -0.11 | (-0.18, -0.03) |  |
| Moncton | 0.04 | (-0.10, 0.17) |  |  | Moncton | 0.18 | (0.04, 0.30) |  |
| Montréal | 0.34 | (0.32, 0.36) |  |  | Montréal | 0.31 | (0.28, 0.33) |  |
| Oshawa | -0.43 | (-0.50, -0.36) |  |  | Oshawa | -0.26 | (-0.33, -0.18) |  |
| Ottawa | 0.20 | (0.15, 0.24) |  |  | Ottawa | 0.44 | (0.41, 0.48) |  |
| Québec | -0.02 | (-0.07, 0.04) |  |  | Québec | 0.20 | (0.15, 0.25) |  |
| Regina | -0.43 | (-0.51, -0.34) |  |  | Regina | -0.37 | (-0.45, -0.27) |  |
| Saint John | -0.19 | (-0.31, -0.06) |  |  | Saint John | 0.21 | (0.08, 0.34) |  |
| Saskatoon | -0.18 | (-0.27, -0.08) |  |  | Saskatoon | -0.02 | (-0.12, 0.08) |  |
| Sherbrooke | -0.37 | (-0.46, -0.26) |  |  | Sherbrooke | -0.13 | (-0.24, -0.02) |  |
| St. Catharines | -0.47 | (-0.53, -0.41) |  |  | St. Catharines | -0.37 | (-0.43, -0.30) |  |
| St. John’s | -0.30 | (-0.40, -0.19) |  |  | St. John’s | 0.01 | (-0.10, 0.12) |  |
| Sudbury | -0.18 | (-0.30, -0.06) |  |  | Sudbury | 0.01 | (-0.11, 0.13) |  |
| Thunder Bay | -0.52 | (-0.61, -0.42) |  |  | Thunder Bay | -0.37 | (-0.47, -0.25) |  |
| Toronto | 0.00 | (-0.02, 0.03) |  |  | Toronto | 0.25 | (0.22, 0.27) |  |
| Vancouver | -0.21 | (-0.24, -0.18) |  |  | Vancouver | 0.09 | (0.05, 0.12) |  |
| Victoria | -0.38 | (-0.45, -0.31) |  |  | Victoria | 0.14 | (0.05, 0.22) |  |
| Windsor | -0.65 | (-0.69, -0.59) |  |  | Windsor | -0.54 | (-0.60, -0.48) |  |
| Winnipeg | -0.36 | (-0.41, -0.31) |  |  | Winnipeg | -0.24 | (-0.29, -0.19) |  |
